# Data on vermicompost effect on the uptake of cadmium from soil by the roots of radish (*Raphanus sativus*)

**DOI:** 10.1016/j.mex.2024.102917

**Published:** 2024-08-22

**Authors:** Ahmad Zarei, Hassan Reza Rokni

**Affiliations:** a Department of Environment Health Engineering, School of public Health, Social Determinates of Health Research Center, Gonabad University of Medical Sciences, Gonabad, Iran; b Student Research Committee, Gonabad University of Medical Sciences, Gonabad, Iran

**Keywords:** Vermicompost, Heavy metal, Cadmium, *Raphanus sativus*, Atomic Absorption Spectroscopy (AAS)

## Abstract

Cadmium is a common environmental heavy metal that is very toxic and carcinogenic for human and other flora and fauna. Therefore, this study is aimed to evaluate the fisibility of vermicompost fertilizer for cadmium uptake from soil by the root of radish (*Raphanus sativus*). For the purpose of the study, four different ratios of one case control, 1 per 1, 1 per 4, 2 per 4, 3 per 4 vermicompost fertilizer per soil with 0, 50000 and 100000 µg/L cadmium concentrations was evaluated. Cadmium in the samples was measured using an Atomic Absorption Spectroscopy (AAS). The results showed that the minimum uptake of cadmium by the plant was observed for 3 per 4 ratio of fertilizer per soil. In addition, results revealed that highest growth rate of *Raphanus sativus* roots occurred in maximum ratio of fertilizer per soil usage (3 per 4). This study showed that vermicompost as a organic fertilizer has a good ability to adsorb cadmium metal from soil. Therfore, vermicompost application can be considered as an inexpensive natural adsorbent in arable land contaminated with cadmium.

• Heavy metals are very toxic and carcinogenic to human and animals.
• Adding organic fertilizer to the soil increases the absorption of heavy metal (cadmium) in the soil and prevents it from entering the food chain.
• The relationship between the concentration of cadmium absorbed by the tuber of radish plant and the percentage of vermicompost added to the soil is presented.

## Graphical abstract

**Figure:**
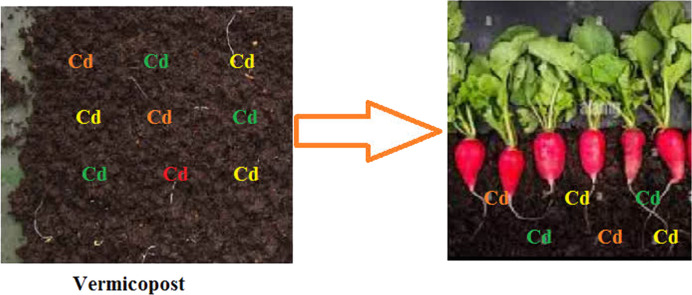

Specifications Table

**Table:** 

Subject area:	Environmental Sciences
More Specific subject area: Name of your method: Name and reference of original method: Resource availability:	Radish Atomic Absorption Spectroscopy (AAS) Standard Methods for the Examination of Water and Wastewater Scale, Vermicompost, Radish seed

## Background

### Introduction

Composting is one of the methods for the final disposal of biodegredable solid wastes. This method, in addition to protecting the environment from the hazards associated with waste materials [[1], [2], [3], [4], [5], [6], [7], [8]], can produce a valuable product applicable as fertilizer in agricultural soils, as well as protecting plants and improving soil quality [[9], [10], [11]]. The main purpose of this study and its results is in line with the above mentioned issue.

Environmental pollution with heavy metals and their transfer to the crops has been a growing global concern. Because, the toxic metals are absorbed and accumulated in different crop species and enter human food chain [[12], [13], [14], [15], [16], [17], [18], [19]].

Most studies on the use of compost fertilizer have been related to its efficiency in plant growth and soil quality improvement [[20], [21], [22]]. While the data from this study are about the role of vermicompost fertilizer as a plant protector against soil contaminants.

The data of present study showed that the organic fertilizer vermicompost has a good ability to adsorb cadmium metal from soil.

The obtained results of this study can provide a basis for future similar studies in relation to protecting crops against heavy metals contamination from soil by vermicompost and other types of composts.

## Method details

### Data description

Table 1 shows the effect of vermicompost organic fertilizer on cadmium uptake by the roots of radish from soil contaminated with cadmium at different treatments concentrations.

**Table 1:** Cadmium uptake by radish plant based on mixture of soil and vermicompost and cadmium concentration.

Run series	Cd concentration (µg/L)	Cd concentration in radish roots (µg/g dried radish root)							Mean	S.D
		1	2	3	4	5	6	7		
**I**	0	3.12	1.98	2.67	2.52	3.23	2.49	1.88	2.55	0.51
	50000	159.85	152.16	147.30	165.31	148.49	155.29	162.40	155.82	6.94
	100000	233.89	283.27	251.86	359.95	320.12	287.40	294.20	299.09	41.77
**II**	0	1.24	1.33	0.94	1.61	1.73	1.13	1.24	1.13	0.27
	50000	50.72	62.06	58.30	64.12	48.30	68.10	56.40	58.28	7.13
	100000	146.32	142.30	135.20	141.14	148.42	158.84	147.61	145.69	7.37
**III**	0	0.76	0.73	0.58	0.51	0.69	0.47	0.68	0.63	0.11
	50000	43.46	37.33	49.40	41.10	38.76	37.45	40.19	41.09	4.24
	100000	70.11	68.39	81.30	64.53	69.12	72.33	63.58	69.90	5.88
**IV**	0	0.29	0.17	0.33	0.18	0.31	0.24	0.40	0.27	0.08
	50000	10.51	9.30	14.20	12.40	8.70	14.63	10.25	11.42	2.34
	100000	28.09	39.03	29.39	22.16	32.37	28.09	34.03	30.45	5.34

### Experimental design

All chemicals used in this study were purchased from Merck (Germany). Before cultivation, samples of soil, water and used fertilizers were prepared and cadmium levels were measured in the laboratory. Cadmium concentrations in them were negligible. Firstly, radish seeds were soaked for 24 hours and then were planted into plastic pots with a height of 25 cm and diameter 15 cm. Each pot contained different ratios of fertilizer to the soil. Then, we sieved soil and vermicompost fertilizer, then in each container we prepared a bed of soil and fertilizer about one kg, the ratio of soil to vermicompost fertilizer was selected with respect to series II- IV. Series I: only the soil, Series II: 75 % soil and 25 % vermicompost, series III: 50 % soil and 50 % vermicompost and series IV: 25 % soil and 75 % vermicompost. Containers were kept under favourable growing conditions. Shortly after radish stated sprouting, we irrigated soils in treatment with solutions containing 0, 50000 and 100000 µg/L of cadmium nitrate. After that, they were irrigated by deionized water until they grew enough. All the process was carried out on the 12 series containing four different concentrations of cadmium and the process was repeated 7 times for each sample. After adding a solution containing cadmium into the soil, we placed samples in a greenhouse under optimum growing conditions for two months. In this study, we focused on the rate of absorption of cadmium by the roots of radish. Therefore, at the end of the growing season, we collected the root of radish to examine levels of cadmium uptake.

### Sample analysis

The concentration of cadmium were measured and analysed according to the Standard Methods for the Examination of Water and Wastewater [23]. For the purpose, we used (Atomic Absorption Spectroscopy (AAS) (Perkin Elmer, analyst 700, USA). All data were analysed using Microsoft Excel software 2016.

## Method validation

The concentration of cadmium analysed in radish samples are shown in Table 1. The results showed that as the ratio of vermicompost organic fertilizer to the soil in the growing environment of the plant increases, the concentration of cadmium in radish decreases. Results given in Table 1 show that by increasing the amount of vermicompost fertilizer in the growing soil of the plant, the accumulation of cadmium in the root of radish decreases. According to the findings of this study, it can be concluded that the use of organic fertilizers in the agricultural soil can effectively absorb cadmium from soil, and by absorbing cadmium, this element does not enter the plant and as a result, it can help food security and protect the food chain from contamination. For the reliability of the samples, each Cd concentration was repeated 7 times as given in Table 1. Limit of detection (LOD) of AAS for cadmium was 1 µg/L. In order to determine the reliability with the amount of cadmium in radish, in addition to the device, we measured with another calibrated device.

This study investigates the effect of organic fertilizer in the soil on the absorption of cadmium to prevent it from entering the food chain. The mentioned method provides a strong framework for the accurate evaluation of the effect of organic materials in the soil on reducing the entry of heavy metals into plants, which may include all types of organic fertilizers. According to the results of this study, vermicompost fertilizer can act as a strong absorber of heavy metals in the soil environment and prevent the entry of heavy metals into plants and thus the human food chain. The validity of this method emphasizes the effectiveness of agricultural organic fertilizers in reducing the concentration of heavy metals in plants and as a result entering the human food chain in real environmental scenarios. This methodology makes a significant contribution to environmental research by providing a systematic approach to understanding and dealing with the effects of environmental disasters, thereby enabling effective environmental protection and management in the face of future challenges.

## CRediT authorship contribution statement

**Ahmad Zarei:** Software, Validation, Writing – review & editing. **Hassan Reza Rokni:** Conceptualization, Methodology, Software, Data curation, Writing – original draft, Visualization, Investigation.

## Declaration of competing interest

The authors declare that they have no known competing financial interests or personal relationships that could have appeared to influence the work reported in this paper.

## Data Availability

The datasets used and/or analysed during the current study available from the corresponding author on reasonable request.
